# 3,3-Ethyl­enedithio-3,3a,4,5,10,10b-hexa­hydro-2*H*-furo[2,3-*a*]carbazole

**DOI:** 10.1107/S1600536809006035

**Published:** 2009-02-25

**Authors:** Nesimi Uludağ, Aslı Öztürk, Tuncer Hökelek, Ümit Işık Erdoğan

**Affiliations:** a Department of Chemistry, Faculty of Technical Education, Mersin University, 33500 Mersin, Turkey; b Department of Physics, Hacettepe University, 06800 Beytepe, Ankara, Turkey; c Department of Chemistry Education, Faculty of Education, Hacettepe University, 06800 Beytepe, Ankara, Turkey

## Abstract

The title compound, C_16_H_17_NOS_2_, consists of a carbazole skeleton with tetra­hydro­furan and dithiol­ane rings. In the indole ring system, the benzene and pyrrole rings are nearly coplanar, forming a dihedral angle of 1.57 (15)°. The cyclo­hexenone and tetra­hydro­furan rings have envelope conformations, while the dithiol­ane ring adopts a twist conformation. In the crystal structure, pairs of weak inter­molecular N—H⋯S hydrogen bonds link the mol­ecules into centrosymmetric dimers with *R*_2_^2^(16) ring motifs. Weak C—H⋯π inter­actions may further stabilize the structure.

## Related literature

For general background, see: Phillipson & Zenk (1980 ▶); Saxton (1983 ▶); Abraham (1975 ▶). For related structures, see: Hökelek *et al.* (1994 ▶, 1998 ▶, 1999 ▶, 2004 ▶, 2006 ▶); Patır *et al.* (1997 ▶); Hökelek & Patır (1999 ▶,2002 ▶); Çaylak *et al.* (2007 ▶). For bond-length data, see: Allen *et al.* (1987 ▶). For hydrogen-bond motifs, see: Bernstein *et al.* (1995 ▶)
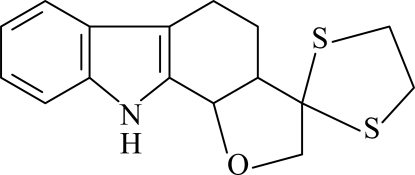


## Experimental

### 

#### Crystal data

C_16_H_17_NOS_2_
*M* *_r_* = 303.43
Orthorhombic, 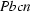

*a* = 21.7617 (5) Å
*b* = 8.4992 (2) Å
*c* = 15.2115 (3) Å
*V* = 2813.47 (11) Å^3^
*Z* = 8
Mo *K*α radiation
μ = 0.37 mm^−1^
*T* = 294 K
0.35 × 0.20 × 0.15 mm

#### Data collection

Enraf–Nonius TurboCAD-4 diffractometer
Absorption correction: ψ scan (North *et al.*, 1968 ▶) *T*_min_ = 0.913, *T*_max_ = 0.944
8196 measured reflections
2289 independent reflections
1105 reflections with *I* > 2σ(*I*)
*R*_int_ = 0.149
3 standard reflections frequency: 120 min intensity decay: 1%

#### Refinement

*R*[*F* ^2^ > 2σ(*F* ^2^)] = 0.041
*wR*(*F* ^2^) = 0.108
*S* = 0.98
2289 reflections
185 parameters
H atoms treated by a mixture of independent and constrained refinement
Δρ_max_ = 0.24 e Å^−3^
Δρ_min_ = −0.23 e Å^−3^

### 

Data collection: *CAD-4 EXPRESS* (Enraf–Nonius, 1994 ▶); cell refinement: *CAD-4 EXPRESS*; data reduction: *XCAD4* (Harms & Wocadlo, 1995 ▶); program(s) used to solve structure: *SHELXS97* (Sheldrick, 2008 ▶); program(s) used to refine structure: *SHELXL97* (Sheldrick, 2008 ▶); molecular graphics: *ORTEP-3 for Windows* (Farrugia, 1997 ▶); software used to prepare material for publication: *WinGX* (Farrugia, 1999 ▶) and *PLATON* (Spek, 2009 ▶).

## Supplementary Material

Crystal structure: contains datablocks I, global. DOI: 10.1107/S1600536809006035/xu2478sup1.cif

Structure factors: contains datablocks I. DOI: 10.1107/S1600536809006035/xu2478Isup2.hkl

Additional supplementary materials:  crystallographic information; 3D view; checkCIF report

## Figures and Tables

**Table 1:** Hydrogen-bond geometry (Å, °)

*D*—H⋯*A*	*D*—H	H⋯*A*	*D*⋯*A*	*D*—H⋯*A*
N10—H10⋯S2^i^	0.81 (4)	2.71 (4)	3.487 (4)	161 (4)
C3*A*—H3*A*⋯*Cg*2^ii^	0.98	2.85	3.725 (4)	149
C4—H4*B*⋯*Cg*1^iii^	0.97	2.79	3.556 (5)	136
C5—H5*A*⋯*Cg*1^ii^	0.97	2.96	3.714 (5)	135

Symmetry codes: (i) 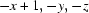; (ii) 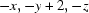; (iii) 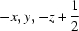. *Cg*1 and *Cg*2 are centroids of the C5b/C6–C9/C9a and C5a/C5b/C9a/N10/C10a rings, respectively.

**Fig. 1.**
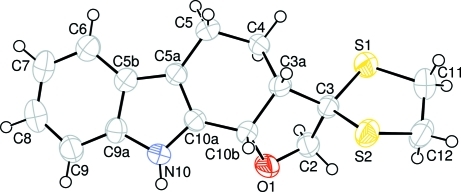
The molecular structure of the title molecule with the atom-numbering scheme. The displacement ellipsoids are drawn at the 50% probability level.

**Fig. 2.**
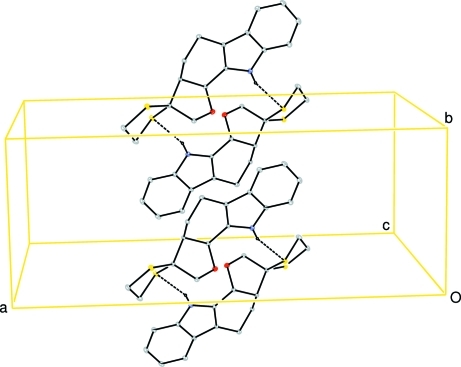
A packing diagram for (I). Hydrogen bonds are shown as dashed lines. H atoms not involved in hydrogen bonding have been omitted for clarity.

## supplementary crystallographic information

### Comment

Tetrahydrocarbazole systems are present in the framework of a number of indole-type alkaloids of biological interest (Phillipson & Zenk, 1980; Saxton, 1983; Abraham, 1975). The structures of tricyclic, tetracyclic and pentacyclic ring systems with dithiolane and other substituents of the tetrahydrocarbazole core, have been the subject of much interest in our laboratory. These include 1,2,3,4-tetrahydrocarbazole-1-spiro-2'-[1,3]dithiolane, (II) (Hökelek *et al.*, 1994), *N*-(2-methoxyethyl)-*N*-{2,3,4,9-tetrahydrospiro[1*H*-carbazole-1, 2-(1,3)dithiolane]-4-yl}benzene-sulfonamide, (III) (Patır *et al.*, 1997), spiro[carbazole-1(2*H*),2'-[1,3]-dithiolan]-4(3*H*)-one, (IV) (Hökelek *et al.*, 1998), 9-acetonyl-3-ethylidene-1,2,3,4-tetrahydrospiro[carbazole-1,2'-[1,3] dithiolan]-4-one, (V) (Hökelek *et al.*, 1999), *N*-(2,2-dimethoxyethyl)-N -{9-methoxymethyl-1,2,3,4-tetrahydrospiro[carbazole-1,2'-[1,3]dithiolan] -4-yl}benzamide, (VI) (Hökelek & Patır, 1999), 3a,4,10,10*b*-tetrahydro-2H -furo[2,3-*a*]carbazol-5(3*H*)-one, (VII) (Çaylak *et al.*, 2007); also the pentacyclic compounds 6-ethyl-4-(2-methoxyethyl)-2,6-methano-5-oxo-hexahydro- pyrrolo(2,3 - d)carbazole-1-spiro-2'-(1,3)dithiolane, (VIII) (Hökelek & Patır, 2002), *N*-(2-benzyloxyethyl)-4,7-dimethyl-6-(1,3-dithiolan-2-yl)-1,2, 3,4,5,6-hexahydro-1,5-methano-2-azocino[4,3-*b*]indol-2-one, (IX) (Hökelek *et al.*, 2004) and 4-ethyl-6,6-ethylenedithio-2-(2-methoxyethyl)-7-methoxy- methylene-2,3,4,5,6,7-hexahydro-1,5-methano-1*H*-azocino[4,3-*b*]indol-3-one, (*X*) (Hökelek *et al.*, 2006). The title compound, (I), may be considered as a synthetic precursor of tetracyclic indole alkaloids of biological interests. The present study was undertaken to ascertain its crystal structure.

In the molecule of the title compound (Fig. 1), the bond lengths (Allen *et al.*, 1987) and angles are within normal ranges. It consists of a carbazole skeleton with tetrahydrofuran and dithiolane rings. The bonds N10—C9a [1.378 (5) Å] and N10—C10*a* [1.371 (5) Å] generally agree with those in compounds (II)-(*X*). In all structures atom N10 is substituted.

An examination of the deviations from the least-squares planes through individual rings shows that rings A (C5b/C6—C9/C9a) and B (C5a/C5b/C9a/N10/C10*a*) are planar. They are also nearly coplanar with a dihedral angle of A/B = 1.57 (15)°. Rings C (C3a/C4/C5/C5a/C10*a*/C10*b*), D (O1/C2/C3/C3a/C10*b*) and E (S1/S2/C3/C11/C12) are not planar. Rings C and D have envelope conformations with atoms C4 and C3 displaced by -0.677 (4) Å (for ring C) and 0.568 (4) Å (for ring D) from the planes of the other ring atoms, respectively. Ring E adopts twisted conformation. Rings C and D have pseudo mirror planes running through atoms C10*a* and C4 (for ring C) and running through atom C3 and midpoint of O1—C10*b* bond (for ring D), as can be deduced from the torsion angles (Table 1).

In the crystal structure, intermolecular N—H···S hydrogen bonds (Table 2) link the molecules into centrosymmetric dimers (Fig. 2) by forming the *R*_2_^2^(16) ring motifs (Bernstein *et al.*, 1995), in which they may be effective in the stabilization of the structure. The weak C—H···π interactions (Table 1) may further stabilize the structure.

### Experimental

For the preparation of the title compound, (I), sodium borohydride (5.00 g, 132.00 mmol) was added to a solution of ethyl 2-(1-oxo-2,3,4,9-tetrahydro-1H -carbazol-2yl)-1,3-dithiolane-2-carboxylate (5.00 g, 13.83 mmol) in THF (50 ml), and stirred at room temperature for 3 h. Then, the reaction mixture was poured into HCl (15%, 100 ml). The crude product was filtered and recrystallized from acetone (yield; 3.2 g, 77%, m.p. 468 K).

### Refinement

H10 atom (for NH) was located in difference synthesis and refined isotropically [N—H = 0.81 (3) Å and *U*_iso_(H) = 0.043 (15) Å^2^]. The remaining H atoms were positioned geometrically, with C—H = 0.93, 0.98 and 0.97 Å for aromatic, methine and methylene H, respectively, and constrained to ride on their parent atoms, with *U*_iso_(H) = 1.2*U*_eq_(C).

### Crystal data

**Table:**

C_16_H_17_NOS_2_	*F*(000) = 1280
*M**_r_* = 303.43	*D*_x_ = 1.433 Mg m^−^^3^
Orthorhombic, *P**b**c**n*	Mo *K*α radiation, λ = 0.71073 Å
Hall symbol: -P 2n 2ab	Cell parameters from 25 reflections
*a* = 21.7617 (5) Å	θ = 9.3–16.7°
*b* = 8.4992 (2) Å	µ = 0.37 mm^−^^1^
*c* = 15.2115 (3) Å	*T* = 294 K
*V* = 2813.47 (11) Å^3^	Prism, colorless
*Z* = 8	0.35 × 0.20 × 0.15 mm

### Data collection

**Table:**

Enraf–Nonius TurboCAD-4 diffractometer	1105 reflections with *I* > 2σ(*I*)
Radiation source: fine-focus sealed tube	*R*_int_ = 0.149
graphite	θ_max_ = 24.3°, θ_min_ = 2.6°
Non–profiled ω scans	*h* = −25→25
Absorption correction: ψ scan (North *et al.*, 1968)	*k* = −9→9
*T*_min_ = 0.913, *T*_max_ = 0.944	*l* = −17→0
8196 measured reflections	3 standard reflections every 120 min
2289 independent reflections	intensity decay: 1%

### Refinement

**Table:**

Refinement on *F*^2^	Primary atom site location: structure-invariant direct methods
Least-squares matrix: full	Secondary atom site location: difference Fourier map
*R*[*F*^2^ > 2σ(*F*^2^)] = 0.041	Hydrogen site location: inferred from neighbouring sites
*wR*(*F*^2^) = 0.108	H atoms treated by a mixture of independent and constrained refinement
*S* = 0.98	*w* = 1/[σ^2^(*F*_o_^2^) + (0.0328*P*)^2^ + 1.8766*P*] where *P* = (*F*_o_^2^ + 2*F*_c_^2^)/3
2289 reflections	(Δ/σ)_max_ < 0.001
185 parameters	Δρ_max_ = 0.24 e Å^−^^3^
0 restraints	Δρ_min_ = −0.23 e Å^−^^3^

### Special details

**Table:**

Geometry. All e.s.d.'s (except the e.s.d. in the dihedral angle between two l.s. planes) are estimated using the full covariance matrix. The cell e.s.d.'s are taken into account individually in the estimation of e.s.d.'s in distances, angles and torsion angles; correlations between e.s.d.'s in cell parameters are only used when they are defined by crystal symmetry. An approximate (isotropic) treatment of cell e.s.d.'s is used for estimating e.s.d.'s involving l.s. planes.
Refinement. Refinement of *F*^2^ against ALL reflections. The weighted *R*-factor *wR* and goodness of fit *S* are based on *F*^2^, conventional *R*-factors *R* are based on *F*, with *F* set to zero for negative *F*^2^. The threshold expression of *F*^2^ > σ(*F*^2^) is used only for calculating *R*-factors(gt) *etc*. and is not relevant to the choice of reflections for refinement. *R*-factors based on *F*^2^ are statistically about twice as large as those based on *F*, and *R*- factors based on ALL data will be even larger.

### Fractional atomic coordinates and isotropic or equivalent isotropic displacement parameters (Å^2^)

**Table:**

	*x*	*y*	*z*	*U*_iso_*/*U*_eq_	
S1	0.32178 (5)	0.10773 (14)	0.23756 (8)	0.0538 (4)	
S2	0.33505 (5)	−0.00621 (14)	0.05706 (9)	0.0538 (3)	
O1	0.48005 (12)	0.0372 (3)	0.1141 (2)	0.0597 (9)	
C2	0.43301 (17)	−0.0004 (5)	0.1754 (3)	0.0499 (12)	
H2A	0.4450	0.0310	0.2343	0.060*	
H2B	0.4250	−0.1126	0.1753	0.060*	
C3	0.37602 (18)	0.0902 (5)	0.1461 (3)	0.0394 (11)	
C3A	0.40456 (17)	0.2447 (4)	0.1109 (3)	0.0391 (11)	
H3A	0.3786	0.2865	0.0637	0.047*	
C4	0.41256 (17)	0.3698 (4)	0.1817 (3)	0.0402 (11)	
H4A	0.3724	0.4058	0.2008	0.048*	
H4B	0.4332	0.3238	0.2320	0.048*	
C5	0.44962 (17)	0.5095 (5)	0.1485 (3)	0.0423 (11)	
H5A	0.4272	0.5632	0.1023	0.051*	
H5B	0.4566	0.5834	0.1961	0.051*	
C5A	0.50957 (18)	0.4512 (4)	0.1140 (3)	0.0375 (10)	
C5B	0.56863 (19)	0.5223 (5)	0.1102 (3)	0.0402 (11)	
C6	0.5923 (2)	0.6705 (5)	0.1333 (3)	0.0487 (12)	
H6	0.5664	0.7483	0.1552	0.058*	
C7	0.6542 (2)	0.6994 (6)	0.1233 (3)	0.0582 (14)	
H7	0.6700	0.7973	0.1384	0.070*	
C8	0.6930 (2)	0.5844 (7)	0.0909 (3)	0.0608 (14)	
H8	0.7347	0.6070	0.0850	0.073*	
C9	0.6721 (2)	0.4373 (5)	0.0671 (3)	0.0562 (13)	
H9	0.6985	0.3607	0.0454	0.067*	
C9A	0.60977 (19)	0.4090 (5)	0.0771 (3)	0.0426 (11)	
C10A	0.51613 (17)	0.3010 (5)	0.0837 (3)	0.0365 (11)	
C10B	0.46569 (17)	0.1854 (4)	0.0715 (3)	0.0401 (11)	
H10B	0.4600	0.1667	0.0085	0.048*	
N10	0.57621 (16)	0.2746 (5)	0.0610 (3)	0.0469 (10)	
H10	0.5901 (17)	0.196 (4)	0.038 (3)	0.043 (15)*	
C11	0.2649 (2)	−0.0299 (5)	0.1985 (3)	0.0658 (14)	
H11A	0.2507	−0.0947	0.2469	0.079*	
H11B	0.2299	0.0269	0.1750	0.079*	
C12	0.2921 (2)	−0.1318 (5)	0.1286 (3)	0.0646 (14)	
H12A	0.2599	−0.1851	0.0961	0.078*	
H12B	0.3188	−0.2106	0.1545	0.078*	

### Atomic displacement parameters (Å^2^)

**Table:**

	*U*^11^	*U*^22^	*U*^33^	*U*^12^	*U*^13^	*U*^23^
S1	0.0516 (7)	0.0527 (7)	0.0570 (8)	−0.0052 (6)	0.0097 (7)	−0.0042 (7)
S2	0.0541 (7)	0.0516 (7)	0.0558 (7)	−0.0125 (7)	−0.0012 (6)	−0.0103 (7)
O1	0.0462 (19)	0.040 (2)	0.093 (3)	0.0094 (14)	0.0183 (18)	0.0122 (18)
C2	0.042 (2)	0.042 (2)	0.065 (3)	0.001 (2)	0.001 (2)	0.008 (3)
C3	0.039 (3)	0.036 (2)	0.042 (3)	−0.003 (2)	−0.001 (2)	−0.001 (2)
C3A	0.038 (2)	0.035 (2)	0.045 (3)	0.0018 (19)	−0.002 (2)	0.000 (2)
C4	0.034 (2)	0.041 (3)	0.047 (3)	0.000 (2)	0.004 (2)	−0.007 (2)
C5	0.048 (3)	0.036 (2)	0.043 (3)	0.001 (2)	−0.001 (2)	−0.004 (2)
C5A	0.040 (3)	0.040 (3)	0.033 (3)	0.000 (2)	0.002 (2)	−0.001 (2)
C5B	0.050 (3)	0.042 (3)	0.029 (2)	−0.003 (2)	−0.001 (2)	0.001 (2)
C6	0.059 (3)	0.049 (3)	0.038 (3)	−0.005 (2)	0.002 (2)	0.002 (2)
C7	0.067 (3)	0.061 (3)	0.046 (3)	−0.025 (3)	−0.002 (3)	0.006 (3)
C8	0.047 (3)	0.082 (4)	0.054 (3)	−0.017 (3)	0.000 (2)	0.009 (3)
C9	0.049 (3)	0.059 (3)	0.061 (3)	−0.003 (3)	0.007 (3)	0.005 (3)
C9A	0.044 (3)	0.046 (3)	0.038 (3)	−0.007 (2)	−0.001 (2)	0.003 (2)
C10A	0.034 (3)	0.040 (3)	0.036 (3)	0.002 (2)	−0.002 (2)	0.002 (2)
C10B	0.042 (3)	0.034 (2)	0.045 (3)	−0.004 (2)	0.004 (2)	0.000 (2)
N10	0.042 (2)	0.040 (2)	0.059 (3)	0.004 (2)	0.011 (2)	−0.006 (2)
C11	0.051 (3)	0.066 (4)	0.080 (4)	−0.017 (3)	0.005 (3)	0.002 (3)
C12	0.070 (3)	0.048 (3)	0.076 (4)	−0.020 (3)	0.004 (3)	−0.007 (3)

### Geometric parameters (Å, °)

**Table:**

S1—C3	1.830 (4)	C5B—C6	1.405 (5)
S1—C11	1.803 (4)	C6—H6	0.9300
S2—C3	1.817 (4)	C7—C6	1.377 (5)
S2—C12	1.788 (4)	C7—C8	1.383 (6)
O1—C2	1.421 (5)	C7—H7	0.9300
O1—C10B	1.450 (4)	C8—H8	0.9300
C2—H2A	0.9700	C9—C8	1.379 (6)
C2—H2B	0.9700	C9—H9	0.9300
C3—C2	1.526 (5)	C9A—C9	1.385 (5)
C3A—C3	1.548 (5)	C9A—C5B	1.408 (5)
C3A—C4	1.523 (5)	C10A—C10B	1.485 (5)
C3A—C10B	1.544 (5)	C10B—H10B	0.9800
C3A—H3A	0.9800	N10—C10A	1.371 (5)
C4—C5	1.522 (5)	N10—C9A	1.378 (5)
C4—H4A	0.9700	N10—H10	0.81 (3)
C4—H4B	0.9700	C11—H11A	0.9700
C5—H5A	0.9700	C11—H11B	0.9700
C5—H5B	0.9700	C12—C11	1.495 (6)
C5A—C5	1.491 (5)	C12—H12A	0.9700
C5A—C10A	1.365 (5)	C12—H12B	0.9700
C5B—C5A	1.421 (5)		
			
C11—S1—C3	98.0 (2)	C5B—C6—H6	120.3
C12—S2—C3	94.1 (2)	C7—C6—C5B	119.3 (4)
C2—O1—C10B	109.5 (3)	C7—C6—H6	120.3
O1—C2—C3	106.3 (3)	C6—C7—C8	120.8 (4)
O1—C2—H2A	110.5	C6—C7—H7	119.6
O1—C2—H2B	110.5	C8—C7—H7	119.6
C3—C2—H2A	110.5	C9—C8—C7	122.1 (4)
C3—C2—H2B	110.5	C9—C8—H8	119.0
H2A—C2—H2B	108.7	C7—C8—H8	119.0
S2—C3—S1	106.7 (2)	C8—C9—C9A	116.9 (4)
C2—C3—S1	110.1 (3)	C8—C9—H9	121.5
C2—C3—S2	112.9 (3)	C9A—C9—H9	121.5
C2—C3—C3A	101.7 (3)	C9—C9A—C5B	122.9 (4)
C3A—C3—S1	116.9 (3)	N10—C9A—C9	130.1 (4)
C3A—C3—S2	108.7 (3)	N10—C9A—C5B	107.1 (4)
C3—C3A—H3A	109.3	O1—C10B—C3A	107.2 (3)
C4—C3A—C3	113.2 (3)	O1—C10B—C10A	111.1 (3)
C4—C3A—H3A	109.3	O1—C10B—H10B	108.9
C4—C3A—C10B	113.8 (3)	N10—C10A—C10B	124.4 (4)
C10B—C3A—C3	101.7 (3)	C3A—C10B—H10B	108.9
C10B—C3A—H3A	109.3	C5A—C10A—N10	109.8 (4)
C3A—C4—H4A	109.3	C5A—C10A—C10B	125.7 (4)
C3A—C4—H4B	109.3	C10A—C10B—C3A	111.9 (3)
C5—C4—C3A	111.8 (3)	C10A—C10B—H10B	108.9
C5—C4—H4A	109.3	C9A—N10—H10	124 (3)
C5—C4—H4B	109.3	C10A—N10—C9A	109.0 (4)
H4A—C4—H4B	107.9	C10A—N10—H10	127 (3)
C4—C5—H5A	109.9	S1—C11—H11A	109.7
C4—C5—H5B	109.9	S1—C11—H11B	109.7
C5A—C5—C4	108.7 (3)	C12—C11—S1	109.8 (3)
C5A—C5—H5A	109.9	C12—C11—H11A	109.7
C5A—C5—H5B	109.9	C12—C11—H11B	109.7
H5A—C5—H5B	108.3	H11A—C11—H11B	108.2
C5B—C5A—C5	131.6 (4)	S2—C12—H12A	110.3
C10A—C5A—C5	121.4 (4)	S2—C12—H12B	110.3
C10A—C5A—C5B	106.8 (4)	C11—C12—S2	107.1 (3)
C6—C5B—C5A	134.6 (4)	C11—C12—H12A	110.3
C6—C5B—C9A	118.0 (4)	C11—C12—H12B	110.3
C9A—C5B—C5A	107.4 (4)	H12A—C12—H12B	108.6
			
C11—S1—C3—S2	15.4 (3)	C5A—C5B—C6—C7	177.8 (4)
C11—S1—C3—C2	−107.4 (3)	C9A—C5B—C6—C7	−0.2 (6)
C11—S1—C3—C3A	137.2 (3)	C5—C5A—C10A—N10	−176.3 (4)
C3—S1—C11—C12	17.0 (4)	C5—C5A—C10A—C10B	7.2 (6)
C12—S2—C3—S1	−36.2 (2)	C5B—C5A—C10A—N10	−0.1 (5)
C12—S2—C3—C2	84.9 (3)	C5B—C5A—C10A—C10B	−176.6 (4)
C12—S2—C3—C3A	−163.1 (3)	C6—C5B—C5A—C5	−2.7 (8)
C3—S2—C12—C11	49.5 (4)	C6—C5B—C5A—C10A	−178.4 (5)
C10B—O1—C2—C3	22.4 (4)	C9A—C5B—C5A—C5	175.4 (4)
C2—O1—C10B—C3A	0.5 (4)	C9A—C5B—C5A—C10A	−0.2 (4)
C2—O1—C10B—C10A	123.0 (4)	C8—C7—C6—C5B	−0.1 (7)
C4—C3A—C3—S1	31.6 (4)	C6—C7—C8—C9	0.2 (7)
C4—C3A—C3—S2	152.4 (3)	C9A—C9—C8—C7	0.0 (7)
C4—C3A—C3—C2	−88.3 (4)	N10—C9A—C5B—C5A	0.5 (4)
C10B—C3A—C3—S1	154.1 (3)	N10—C9A—C5B—C6	179.0 (4)
C10B—C3A—C3—S2	−85.1 (3)	C9—C9A—C5B—C5A	−178.1 (4)
C10B—C3A—C3—C2	34.2 (4)	C9—C9A—C5B—C6	0.4 (6)
C3—C3A—C4—C5	171.0 (3)	N10—C9A—C9—C8	−178.5 (4)
C10B—C3A—C4—C5	55.6 (4)	C5B—C9A—C9—C8	−0.3 (6)
C4—C3A—C10B—O1	99.6 (4)	N10—C10A—C10B—O1	55.3 (5)
C3—C3A—C10B—O1	−22.4 (4)	N10—C10A—C10B—C3A	175.1 (4)
C4—C3A—C10B—C10A	−22.4 (5)	C5A—C10A—C10B—O1	−128.7 (4)
C3—C3A—C10B—C10A	−144.4 (3)	C5A—C10A—C10B—C3A	−9.0 (6)
S1—C3—C2—O1	−160.3 (3)	C9A—N10—C10A—C5A	0.4 (5)
S2—C3—C2—O1	80.6 (4)	C9A—N10—C10A—C10B	176.9 (4)
C3A—C3—C2—O1	−35.7 (4)	C10A—N10—C9A—C5B	−0.5 (5)
C3A—C4—C5—C5A	−55.3 (4)	C10A—N10—C9A—C9	177.8 (4)
C5B—C5A—C5—C4	−149.7 (4)	S2—C12—C11—S1	−44.2 (4)
C10A—C5A—C5—C4	25.4 (5)		

### Hydrogen-bond geometry (Å, °)

**Table:**

*D*—H···*A*	*D*—H	H···*A*	*D*···*A*	*D*—H···*A*
N10—H10···S2^i^	0.81 (4)	2.71 (4)	3.487 (4)	161 (4)
C3A—H3A···Cg2^ii^	0.98	2.85	3.725 (4)	149
C4—H4B···Cg1^iii^	0.97	2.79	3.556 (5)	136
C5—H5A···Cg1^ii^	0.97	2.96	3.714 (5)	135

Symmetry codes: (i) −*x*+1, −*y*, −*z*; (ii) −*x*, −*y*+2, −*z*; (iii) −*x*, *y*, −*z*+1/2.
